# Type 2 diabetes mellitus increases the risk of circumcision among men aged between 30 and 69 years using a nationwide population-based dataset in Taiwan: a five-year follow-up study

**DOI:** 10.1186/s12894-023-01392-6

**Published:** 2024-01-03

**Authors:** Yun-Rui Wu, Yi-Horng Lai, Chung-Cheng Wang

**Affiliations:** 1 Department of Urology, College of Medicine, En Chu Kong Hospital, National Taiwan University, 399 Fu-hsing Road, Sanxia District, New Taipei City, 23702 Taiwan; 2 Department of Healthcare Administration, Asia Eastern University of Science and Technology, New Taipei City, 22061 Taiwan; 3 Department of Biomedical Engineering, Chung Yuan Christian University, Taoyuan, 32023 Taiwan

**Keywords:** Diabetes Mellitus, Circumcision, Epidemiology

## Abstract

**Background:**

Diabetes is an important factor in the development of penile inflammation. We studied whether type 2 diabetes (DM), with/without hypertension and hyperlipidemia increased the risk of circumcision among men aged between 30 and 69 using a population-based dataset in Taiwan during a 5-year follow-up period.

**Methods:**

The research data in this study were obtained from Taiwan’s National Health Insurance Research Database between 1997 and 2010. We identified 23,197 patients who had a new diagnosis of DM and randomly matched 115,985 subjects as controls. We observed whether circumcision was the treatment after a new DM diagnosis. The initial step involved analyzing the data using Poisson regression analysis. To address potential confounding factors, this study employed propensity score matching based on three variables. Additionally, a Cox regression with a Gamma frailty was utilized to compare outcomes between different groups.

**Results:**

Poisson regression analysis showed that DM (RR = 1.75, 95CI = 0.10 ~ 1.22), but not hypertension (RR = 1.14, 95CI=-0.44 ~ 0.70), hyperlipidemia (RR = 0.94, 95CI=-0.66 ~ 0.53), or age (RR = 0.83, 95CI=-0.43 ~ 0.62), had an impact on circumcision treatment. Cox regression with a frailty model found that DM was a risk factor associated with circumcision (HR = 2.31, 95% CI = 1.74 ~ 3.06, *p*-value < 0.01), whereas no significant difference was noted between circumcision and hypertension (HR = 1.10, 95% CI = 0.80 ~ 1.51), hyperlipidemia (HR = 1.05, 95% CI = 0.79 ~ 1.40), or age (HR = 1.00, 95% CI = 0.99 ~ 1.02).

**Conclusions:**

Type 2 diabetes mellitus, but not hypertension, hyperlipidemia or age increases the risk of circumcision in men aged between 30 and 69 years.

**Supplementary Information:**

The online version contains supplementary material available at 10.1186/s12894-023-01392-6.

## Background

Male circumcision is the most common surgical procedure because routine infant circumcision is performed in many countries for religious and cultural reasons [1]. The estimated country-specific prevalence of circumcision varies greatly in different countries, e.g., 99.8% in Afghanistan, 99.7% in Iran, 71.2% in the United States, and 0.2% in Vietnam [2]. This discrepancy reflects the controversy regarding whether circumcision is mandatory. Proponents claim benefits such as improved local hygiene and decreased risk of urinary tract infection, sexually transmitted diseases and penile and cervical cancer, whereas opponents emphasize procedure-related complications and insult to the autonomy of the children [3]. The European Association of Urology (EAU) guidelines on pediatric urology recommend three conditions for circumcision: balanitis xerotica obliterans (BXO), phimosis refractory to treatment, and if patient/caregivers prefer circumcision for symptomatic phimosis [4].

Several epidemiological studies have suggested that type 2 diabetes mellitus (T2DM) is associated with BXO and refractory phimosis. Using a nationwide population-based study, Wang et al. demonstrated that T2DM was a risk factor associated with penile inflammatory disorders (HR = 1.42, 95% CI = 1.27 ~ 1.68, p < 0.01) [5]. In the UK General Practice Research Database, Hirji et al. showed that diabetes mellitus (DM) patients had a relative risk of 2.85 (2.39 ~ 3.39) for the incidence of balanitis compared to patients without diabetes [5]. In addition, in an outpatient setting, Fakjian et al. found that uncircumcised male patients with T2DM had a high (35%) prevalence of symptomatic phimosis [6]. Ke et al. proposed that balanoposthitis with a volcano-like appearance might be the first clinical presentation of undiagnosed DM [7]. In the fifty- to sixty-year-old age group, 83.3% of circumcised patients had diabetes [8]. This evidence suggests that DM increases the risk of penile inflammatory diseases and might increase the diabetes-related need for circumcision in adult males. Interestingly, we recently asked ChatGPT “Does diabetes mellitus increase the risk of circumcision?”, and the answer was “No”.

Triple H (hyperglycemia, hypertension, and hyperlipidemia) usually coexists in patients with metabolic syndromes. The association between triple H and adult circumcision has rarely been investigated. Thus, we studied whether T2DM, with/without hypertension and hyperlipidemia increased the risk of circumcision among men aged between 30 and 69 using a population-based dataset in Taiwan during a 5-year follow-up period.

## Materials and methods

### Study design

We conducted a nationwide cohort study of 1 million patients from Taiwan’s National Health Insurance Research Database (NHIRD) between 1997 and 2010 [9]. We included men aged between 30 and 69 years because type 2 DM is not common in aged ≤ 30 years in Taiwan. In addition, men aged ≤ 30 years might undergo circumcision due to cosmetic or other factors. Second, men aged ≥ 70 years have more co-morbidities which may be confounding factors. Patients with type 1 DM were also excluded from the study because of different pathophysiology mechanisms between type 1 and type 2 DM.

To identify patients with newly diagnosed DM (ICD-9-CM code 250) between 1998 and 2005, this study excluded patients who had a new DM diagnosis before 1998 or after 2005 and patients who underwent circumcision treatment before a DM diagnosis. We observed whether circumcision was the treatment after a new DM diagnosis. A total of 191,601 out of 1 million patients qualified for this study, and 23,197 male patients with newly diagnosed DM between 1998 and 2005 were identified. We decided the time scale of 5 years based on our previous study which showed type 2 DM increased the risk of penile inflammatory disorders in men in a 5-year follow-up study [10]. Among the 23,197 patients with type 2 DM during the follow-up period that ended in December 2010, sixty-nine patients underwent circumcision.

The Institutional Review Board and Ethics Committee of En Chu Kong Hospital, Taipei, Taiwan, approved this study (Identifier ECKIRB1110501, the approval date: 22-Jun-2022) and waived the informed consent requirement because the NHIRD consists of deidentified secondary data released to the public for research purposes. All methods performed in studies involving human participants were in accordance with the ethical standards of the institutional research committee and with the Helsinki Declaration. Results are reported in accordance with Strengthening the Reporting of Observational Studies in Epidemiology (STROBE) reporting guideline [11].

The primary outcome observed in this study was circumcision for phimosis (NHI diagnosis code 50,020 C). The occurrence of these diseases during the follow-up period was defined as at least one outpatient visit or one impatient admission with a diagnosis of comorbidity with hypertension (ICD-9-CM codes 401–405) and hyperlipidemia (ICD-9-CM code 272).

**Fig. 1:**
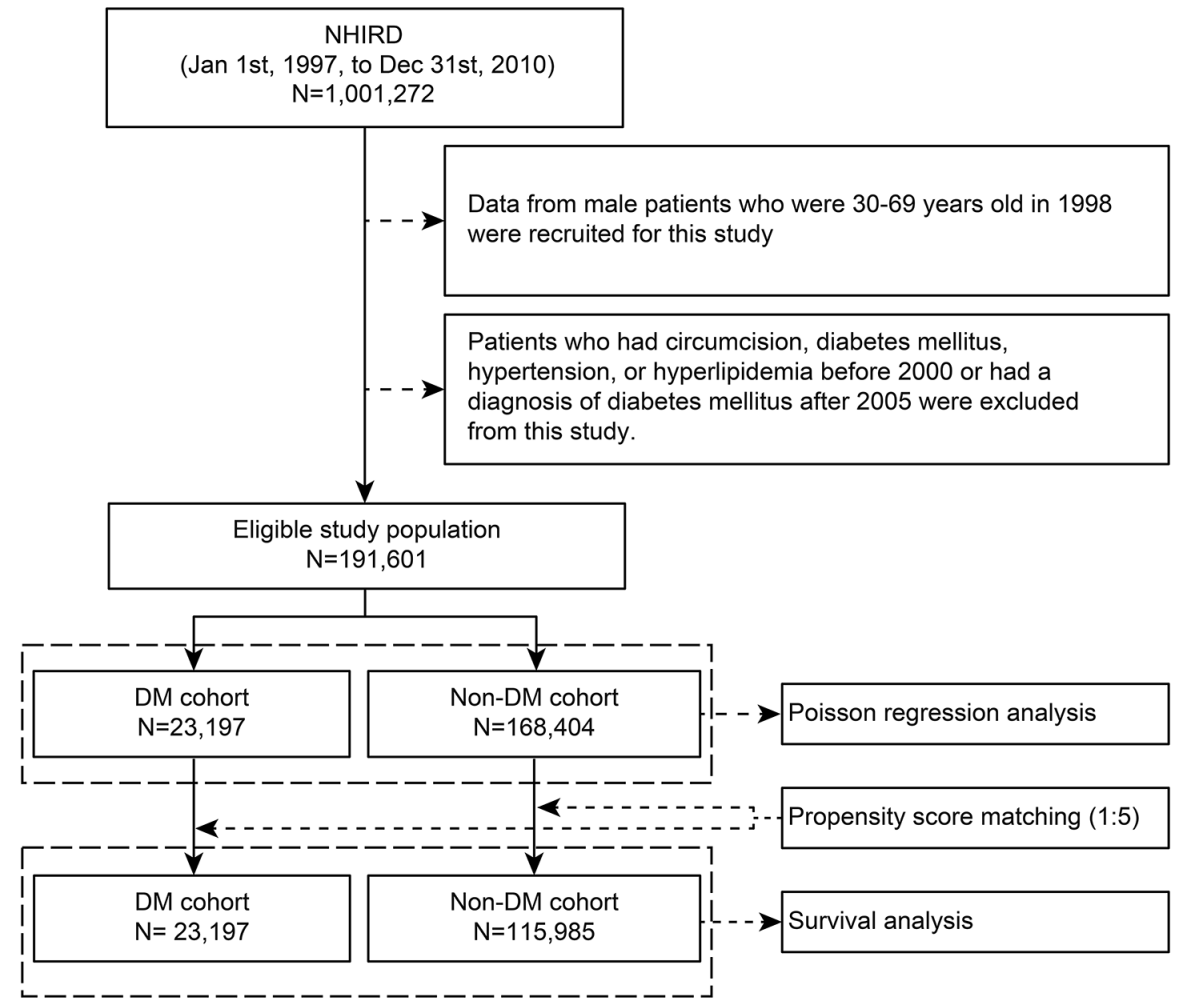
Flowchart depicting the process of identifying the study participants and their classification into the diabetes mellitus (DM) and non-DM groups

After the above processes, the participants who met the inclusion and exclusion criteria were classified into the DM group. The other patients were classified into the non-DM group. A detailed schematic of the process is depicted in Fig. 1.

### Data analysis

The research data were first analyzed with Poisson regression analysis and then by Cox regression with a frailty model after propensity score matching. The results of these two different analyses were compared, and all data analyses were performed using R 4.2.0 with the survival package [12].

Poisson regression analysis was used to determine the incidence of circumcision treatment in both cohorts. The DM and non-DM cohorts were compared by using Poisson regression analysis with adjustments for comorbidity with hypertension, comorbidity with hyperlipidemia, and age.

Propensity score matching was used to reduce selection bias due to measurable confounding variables between the exposed and nonexposed groups and to make this study similar to a randomized trial [13]. Conducting propensity score matching could make the two groups have a similar probability of exposure to DM. Logistic regression and the previously mentioned covariates were used to calculate a propensity score for each patient. Then, patients in the two groups were matched by propensity score through a one-to-five greedy matching process [14]. Finally, Cox regression with Gamma frailty was used to re-assess the results. The frailty model allows one to add a simple random effects term to a Cox model. Frailty model was applied in this study.

## Results

The demographic characteristics of the study participants in each group are presented in supplementary Table 1. A total of 191,601 patients were included in this study. Among them, three hundred ninety-nine (0.21%) underwent circumcision treatment, and 191,202 (99.79%) did not undergo circumcision treatment during the follow-up period. A total of 23,197 patients (12.11%) had T2DM, 28,499 patients (14.87%) had comorbid hypertension, and 47,180 patients (24.62%) had comorbid hyperlipidemia.

Among the 23,197 patients with DM during the follow-up period that ended in December 2010, 69 had circumcision treatment, 4,220 developed hypertension, and 5,351 developed hyperlipidemia (supplementary Table 2). Compared with the non-DM cohort, the DM cohort exhibited higher prevalence rates of circumcision (0.30% vs. 0.20%, respectively), hypertension (18.19% vs. 14.42%, respectively), and hyperlipidemia (23.07% vs. 24.84%, respectively) (all *p* values < 0.001).

### Poisson regression analysis results

Based on the results of Poisson regression, Table 1 shows the relationship between circumcision treatment and DM, hypertension, hyperlipidemia, and age. DM (RR = 1.75, 95CI = 0.10 ~ 1.21), but not hypertension (RR = 1.14, 95CI= -0.44 ~ 0.70), hyperlipidemia (RR = 0.94, 95CI= -0.66 ~ 0.52), or age (RR = 0.83, 95CI= -0.43 ~ 0.62), had an impact on circumcision treatment. Men with DM has a 1.75-fold higher likelihood of developing circumcision treatment compared to men without DM.

**Table 1:** Poisson regression model for circumcision

Variables	Est.	S. E.	*z* value	Pr (>|z|)	RR^a^
(Intercept)	-5.56	0.21	-26.03	< 0.001	0.003
Diabetes mellitus	0.56	0.14	4.04	< 0.001	1.75
Hypertension	-0.07	0.20	-0.33	0.74	0.94
Hyperlipidemia	0.13	0.16	0.80	0.43	1.14
Age in 1998	-0.18	0.06	-3.32	< 0.001	0.83

a. Risk ratio

### Results of cox regression with a frailty model

Individual characteristics and the initial health status in the DM and non-DM groups are shown in Table 2. After using propensity score matching for the DM and non-DM groups for comorbid hypertension, hyperlipidemia, and age in 1998 with a 1:5 ratio, there were 23,197 participants included in the DM group and 115,985 participants included in the non-DM group (supplementary Table 3).

**Table 2:** The results of extended Cox regression with a frailty model for circumcision and type 2 diabetes mellitus and associated covariates

Variables	Crude^b^			Adjusted^c^		
	HR^a^	(95% CI^d^)	*p*-value	HR^a^	(95% CI^d^)	*p*-value
DM	2.31	(1.74, 3.06)	< 0.001	2.31	(1.74, 3.06)	< 0.001
Comorbid hypertension	1.10	(0.80, 1.51)	0.95	-	-	
Comorbid hyperlipidemia	1.05	(0.79, 1.40)	0.64	-	-	
Age in 1998	1.00	(0.99, 1.02)	0.76	-	-	

*p < 0.05

a. Hazard ratio

b. Crude HR, relative hazard ratio

c. Adjusted HR: Multivariable analysis including DM, hypertension, hyperlipidemia, and age in 1998

d. 95% confidence interval

Table 2 shows the results of the univariable and multivariable Cox regression analyses with a frailty model. Patients with diabetes had a significantly increased risk of circumcision treatment compared to non-DM patients (adjusted hazard ratio (HR) = HR = 2.31, 95% CI = 1.74 ~ 3.06). However, hypertension (crude HR = 1.10, 95% CI = 0.80 ~ 1.51), hyperlipidemia (crude HR = 1.05, 95% CI = 0.79 ~ 1.40), and age (crude HR = 1.00, 95% CI = 0.99 ~ 1.02) were not correlated with circumcision treatment. Men with DM is 2.31 times more likely to undergo circumcision than men without DM. These results were consistent with the results of the Poisson regression analysis.

**Fig. 2:**
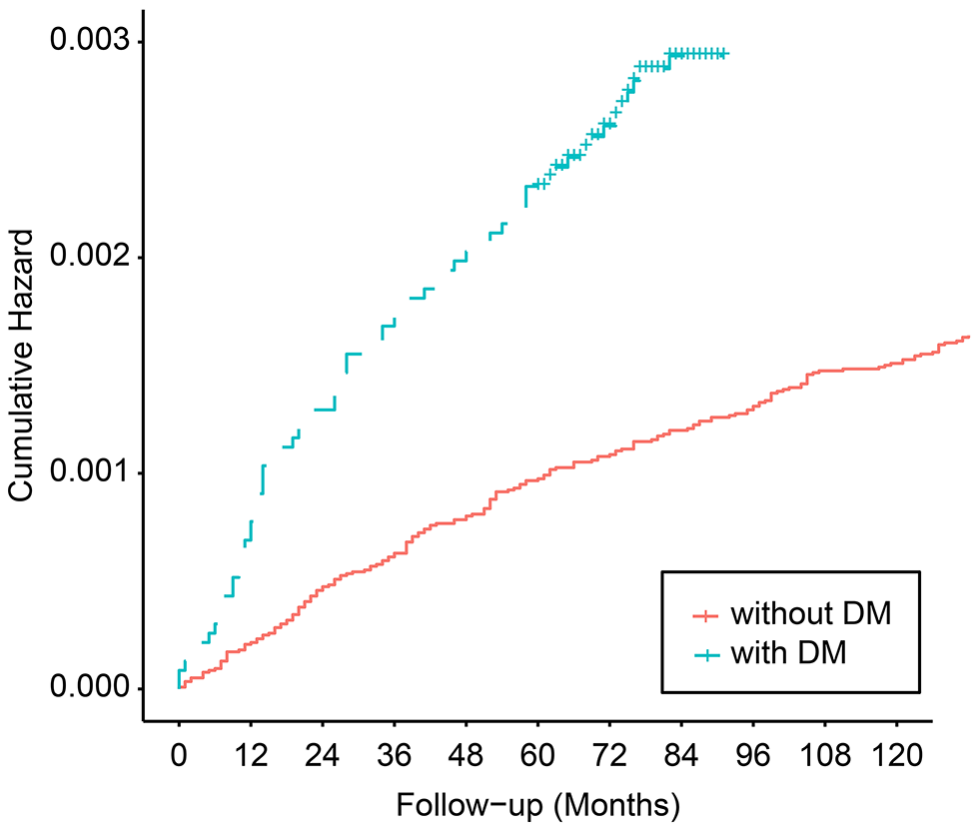
Cumulative hazard curve for circumcision in men with and without diabetes aged between 30 to 69 years. (log-rank test, p < 0.001)

The mean lengths of time for follow-up were 98.1 ± 21.6 months for the DM cohort and 131.9 ± 3.3 months for the non-DM cohort. Figure 2 outlines the results of the Kaplan‒Meier and log-rank tests. The log-rank test showed that patients with DM had a significantly higher incidence of circumcision than those without DM (*p* value < 0.001). The median cumulative incidence of circumcision treatment was 0.1% higher in the DM cohort than in the non-DM cohort in the 5th year of follow-up and 0.2% higher in the DM cohort than in the non-DM cohort at the end of the 10th year of follow-up.

## Discussion

To our knowledge, this study is the first to investigate the risk of circumcision treatment in a large cohort of men aged 30 to 69 years with or without DM, hyperlipidemia, and hypertension during a 5-year follow-up period. This study demonstrated that T2DM, but not hypertension or hyperlipidemia, was associated with adult circumcision. A previous large longitudinal cohort study revealed that penile inflammatory disorders were more common among males with T2DM [6, 10]. In addition, recurrent balanoposthitis and balanitis xerotica obliterans (BXO) are considered surgical indications for circumcision [1]. Taking these study results together, the findings of this study provided direct evidence that T2DM was associated with an increased risk of circumcision among adult men.

From the point of view of preventive medicine, circumcision can reduce the risk of heterosexual HIV transmission and genital infection in men. From the point of view of Jewish law, circumcision is considered a fundamental commandment of the faith. However, the main medical indications for circumcision are pathological phimosis and recurrent balanoposthitis [1]. Pathological phimosis in adults can be easily distinguished from physiological phimosis, which is a normal preputial adhesion between the prepuce and glans penis in young boys. The specific macroscopic features of diabetic balanitis include acquired phimosis, preputial fissures and a volcano-like appearance of the prepuce [7, 15]. Thus, physicians should attempt early detection of diabetes-associated penile inflammatory disorders. In cases refractory to conservative treatment, circumcision might be suggested to prevent further complications.

Although many advantages could be achieved by circumcision, the possibility of complications and long-term dissatisfaction should not be ignored. A systematic review reported up to 47 specific complications arising from male circumcision [16]. The report suggested that experienced physicians who practice in sterile settings had better outcomes with few complications. Another meta-analysis found that the overall complication risk of male circumcision was 3.84% (95% confidence interval 3.35–4.37) [17]. A higher complication rate was noted in therapeutic circumcision than in nontherapeutic circumcision (7.47% versus 3.34%, p < 0.05). In addition, several studies have demonstrated that DM confers an increased risk of short- and long-term morbidity and mortality among patients undergoing surgical treatment, e.g., colectomy and hernia repair [18, 19]. However, only one study on adult circumcision demonstrated that obesity (45.0% versus 22.1%, p = 0.02), but not DM (60.0% versus 44.3%, p > 0.05), hypertension (55.0% versus 57.1%, p > 0.05) or hyperlipidemia (50.0% versus 47.8%, p > 0.05), increased the risk of wound complications [20]. Since obesity often coexists with triple H, it is necessary to investigate the association of DM and complications of circumcision in the future. Recently, a meta-analysis showed that compared with traditional circumcision, circumcision using disposable circumcision suture devices had the advantages of a shorter operative time, shorter wound healing time, less blood loss and better cosmetic penile appearance [21]. Physicians might recommend novel surgical technology to diabetic patients needing circumcision to obtain better outcomes.

Many studies have suggested that triple H or metabolic syndrome is associated with the morbidity or mortality of cardiovascular diseases, cancer, complications after surgery and infectious diseases, including genital and urinary tract infections [22–24]. However, the study disclosed that only DM, but not hypertension or hyperlipidemia, was a risk factor for circumcision in adult males. The findings from the study echoed those from a large epidemiological survey that revealed that type 2 DM, but not hypertension or hyperlipidemia, was associated with an increased risk of penile inflammatory diseases in men between 30 and 49 years of age [10]. Since compared to non-DM controls, only DM patients had more cases of balanoposthitis, we presumed that the medical need for circumcision would increase among DM patients.

Our study had some limitations. First, diagnosis and treatment rely on administrative claims data and ICD codes but not pathological reports. Diabetic patients might undergo circumcision due to cosmetic or other factors but not genital infection. Second, the database might not represent patients with balanitis. Some patients with mild balanitis might not seek medical help because they think the disease is shameful and not bothersome until repeated infection or an inability to have sex occurs. Third, the study population mainly consisted of people of Taiwanese ethnicity who have an extremely low prevalence of neonatal circumcision. It is unclear whether the results can be applied to other ethnic populations or countries with a high prevalence of neonatal circumcision. Nevertheless, the strength of this study was the use of a nationwide population-based dataset that provided a sufficient sample size and statistical power to investigate the association between diabetes mellitus and circumcision.

## Conclusion

Type 2 diabetes mellitus, but not hypertension, hyperlipidemia or age, was associated with an increased risk of circumcision treatment among men aged between 30 and 69 years.

### Electronic supplementary material

Below is the link to the electronic supplementary material.

Supplementary Material 1

## Data Availability

The data that support the findings of this study are available on request from the corresponding author, [CCW]. Data available on request due to privacy restrictions.

## Abbreviations

T2DM: Type 2 diabetes mellitus
DM: Diabetes mellitus
BXO: Balanitis xerotica obliterans
CI: Confidence interval
OR: Odds ratio
HR: Hazard ratio
NHIRD: National Health Insurance Research Database
