# Supplementary material for: Type 2 diabetes mellitus increases the risk of circumcision among men aged between 30 and 69 years using a nationwide population-based dataset in Taiwan: a five-year follow-up study
Source: BMC Urol. 2024 Jan 3;24:3. doi: 10.1186/s12894-023-01392-6 (PMC10765904; doi:10.1186/s12894-023-01392-6)
Supplement: Supplementary file 1 — Supplementary Material 1 [file 12894_2023_1392_MOESM1_ESM.docx]

**Supplementary table 1.** The case number of circumcision, diabetes mellitus, hypertension and hyperlipidemia of the participants in this study (N=191,601).

| Characteristics |  | Case number | % |
| --- | --- | --- | --- |
| Circumcision | Yes | 399 | 0.21 |
|  | No | 191,202 | 99.79 |
| Diabetes mellitus | Yes | 23,197 | 12.11 |
|  | No | 168,404 | 87.89 |
| Comorbidity-Hypertension | Yes | 28,499 | 14.87 |
|  | No | 163,102 | 85.13 |
| Comorbidity-Hyperlipidemia | Yes | 47,180 | 24.62 |
|  | No | 144,421 | 75.38 |
| Age in 1998, mean (SD), years |  | 44.4 (10.4) |  |

SD: standard deviation

**Supplementary table 2.** Comparison of circumcision, hypertension, and hyperlipidemia in the diabetes mellitus (DM) and non-DM cohorts (N=191,601)

|  |  | DM  N=23197  n (%) | Non-DM  N=168404  n (%) | p-value |
| --- | --- | --- | --- | --- |
| Circumcision | Yes | 69 (0.30) | 330 (0.20) | 0.001^a^ |
|  | No | 23,128 (99.7) | 168,074 (99.8) |  |
| Comorbid  hypertension | Yes | 4,220 (18.2) | 24,279 (14.4) | <0.001^a^ |
|  | No | 18,977 (81.8) | 144,125 (85.6) |  |
| Comorbid  hyperlipidemia | Yes | 5,351 (23.1) | 41,829 (24.8) | <0.001^a^ |
|  | No | 17,846 (76.9) | 126,575 (75.2) |  |
| Age, mean (SD) |  | 51.226 (10.0) | 43.377 (10.1) | <0.001^b^ |

a. Chi-square test

b. Independent two-sample *t* test.

SD: standard deviation

**Supplementary table 3.** Comparisons of circumcision, hypertension, hyperlipidemia and age in patients with/without DM after stratification by propensity score matching (1:5)

|  |  | DM  (n=23,197) | Non-DM  (n=115,985) | p-value |
| --- | --- | --- | --- | --- |
| Circumcision | Yes | 69 | 183 | 0.17^a^ |
|  | No | 23,128 | 115,802 |  |
| Comorbid  hypertension | Yes | 4,220 | 20,416 | 0.23^a^ |
|  | No | 18,977 | 95,569 |  |
| Comorbid  hyperlipidemia | Yes | 5,351 | 27,678 | 0.73^a^ |
|  | No | 17,846 | 88,307 |  |
| Age, mean (SD) |  | 50.0 (10.4) | 49.4 (9.1) | 0.46^b^ |

a. Chi-square test

b. Independent two-sample *t* test.

DM, diabetes mellitus.
